# Spatiotemporal Variability of Soil Nitrogen in Relation to Environmental Factors in a Low Hilly Region of Southeastern China

**DOI:** 10.3390/ijerph15102113

**Published:** 2018-09-26

**Authors:** Shan He, Hailun Zhu, Amir Reza Shahtahmassebi, Lefeng Qiu, Chaofan Wu, Zhangquan Shen, Ke Wang

**Affiliations:** 1 Institute of Agriculture Remote Sensing and Information Technology Application, College of Environment and Natural Resource, Zhejiang University, Hangzhou 310058, China; heshan33@zju.edu.cn (S.H.); zhuhailun@zju.edu.cn (H.Z.); Amir511@zju.edu.cn (A.R.S.); zhqshen@zju.edu.cn (Z.S.); 2 Institute of Land and Urban-Rural Development, Zhejiang University of Finance and Economics, Hangzhou 310018, China; qiulefeng@zufe.edu.cn; 3 College of Geography and Environmental Sciences, Zhejiang Normal University, Jinhua 321004, China; cfwdh@zjnu.edu.cn

**Keywords:** soil total nitrogen, digital soil mapping, spatiotemporal distribution, mountainous region

## Abstract

Soil total nitrogen (TN) plays a major role in agriculture, geochemical cycles and terrestrial ecosystem functions. Knowledge regarding the TN distribution is crucial for the sustainable use of soil resources. This paper therefore aims to characterize the spatiotemporal distribution of soil TN and improve the current understanding of how various factors influence changes in TN. Natural characteristics and remote sensing (RS) variables were used in conjunction with the random forest (RF) model to map the TN distribution in a low hilly region of southeastern China in 1979, 2004 and 2014. The means and changes of TN in different geographic regions and farmland protection regions were also analyzed. The results showed that: (1) the TN showed an increasing trend in the early periods and exhibited a decreasing trend from 2004 to 2014; (2) the geographic and RS variables played more important roles in predicting TN distribution than did the other variables; and (3) changes in the fertilization and crop planting structure caused by soil testing and formulated fertilization techniques (STFFT—Soil Testing and Formulated Fertilization Techniques) as well as farmland protection policies influenced the spatiotemporal variability of TN. Evidently, more attention should be focused on improving the quality and soil fertility in the surrounding low mountainous areas.

## 1. Introduction

Soil represents the foundation for agricultural activities, and it is directly related to the safety of agricultural products [1]. Accordingly, reasonable soil management contributes to the sustainable development of agriculture, increases human welfare, and sustains economic and social development [2]. Soil total nitrogen (TN) constitutes one of the most important indicators used to evaluate whether such soil management is reasonable [3] because soil TN plays a major role in agriculture (e.g., controlling soil fertility and plant productivity), geochemical cycles and terrestrial ecosystem functions [4].

The aforementioned advantages of soil TN in agriculture have prompted the excessive use of nitrogen (N) fertilizer by farmers. However, increasing the amount of soil TN presents considerable problems for the environment, climate, and natural resources in addition to human health [5,6]. Such an increase in soil TN could also reduce plant N use efficiency, which could suppress further increases in crop yields [7]. To reduce irrational fertilization, the Chinese government has introduced soil testing and formulated fertilization techniques (STFFT) to vast areas of mainland China (Chinese territory) since 2000 [8]. Furthermore, because large amounts of high-quality cultivated land have been lost to urbanization and contamination, the Chinese government has implemented a series of measures (e.g., land consolidation and the delineation of permanent basic farmland areas and grain production areas) to protect current high-quality arable land and promote the comprehensive conditions of arable land [9]. The aforementioned various field management methods may have either directly or indirectly affected the spatiotemporal distribution of soil TN in recent decades [10]. Consequently, increases in interest and debate have arisen regarding whether agricultural policy might influence changes in soil TN.

A wealth of literature is available that presents measurements of soil TN using digital soil mapping (DSM) techniques based on the integration of remotely sensed imagery with a geographic information system (GIS). These studies on DSM have employed two types of data mining techniques: aspatial techniques (e.g., boosted regression tree (BRT) [11], artificial neural network (ANN) [12], and random forest (RF) [13] models) and spatial techniques (e.g., regression kriging (RK) [14] and geographically weighted regression (GWR) [15] analysis). In terms of data, both hyperspectral data and field sampling data have been used in DSM models [16]. However, although these studies have offered intelligent and informative results, several problems still exist, and those issues are outlined below.

(1) No systematic methodology exists that can be used to analyze the spatiotemporal changes in soil TN in conjunction with agricultural protection policies. Timely monitoring of the spatial distribution of soil TN can provide information on both the location and the amount of N in the soil; thus, explicitly linking changes in soil TN to agricultural protection regulations could be useful for understanding the dynamic patterns of N in soil [10] and for assessing the degree to which the Chinese STFFT decrease or increase soil TN in agricultural land.
(2) Most DSM studies have focused on integrating hyperspectral data with field surveys and environmental parameters to monitor changes in soil TN [17]. However, archives of satellite sensor imagery such as Landsat data also include useful information that has not been fully exploited.
(3) While DSM has been hailed as a very useful technique for assessing and monitoring soil TN [11], its applications thus far have been restricted to flat lands, partly because of the easy accessibility of such regions and partly because of the less complex environment. In contrast, the challenging environments of mountainous regions, which exhibit nonlinear relationships between soil properties and environmental predictors, have prevented researchers from analyzing the spatiotemporal patterns of soil TN therein [17], especially in terms of the relationship between soil TN and agricultural protection policies.

This paper aims to characterize the spatiotemporal distribution of soil TN and improve the general understanding of how various factors influence changes in soil TN. To do so, this study contributes to two crucial aspects in the context of digital soil TN mapping. First, we propose a DSM framework using aspatial data mining and geospatial analysis to quantify the spatiotemporal distribution of soil TN in mountainous environments; then, we ascertain the key auxiliary covariates and their varied effects in predicting soil TN. Second, we systematically link the observed variations to agricultural protection policies. Fuyang District located in Zhejiang Province, China, was selected as a typical example of a mountainous region to conduct this research. We analyzed the variation in soil TN in this district over two periods, i.e., before (1979–2004) and after (2004–2014) the implementation of the Chinese STFFT. We selected 2004 as the time node because our soil sampling survey was performed in 2004. Consequently, through performing a detailed investigation into the regional soil TN, the potential for future protection regulations was more clearly defined.

## 2. Materials and Methods

### 2.1. Site Description

**Figure 1:**
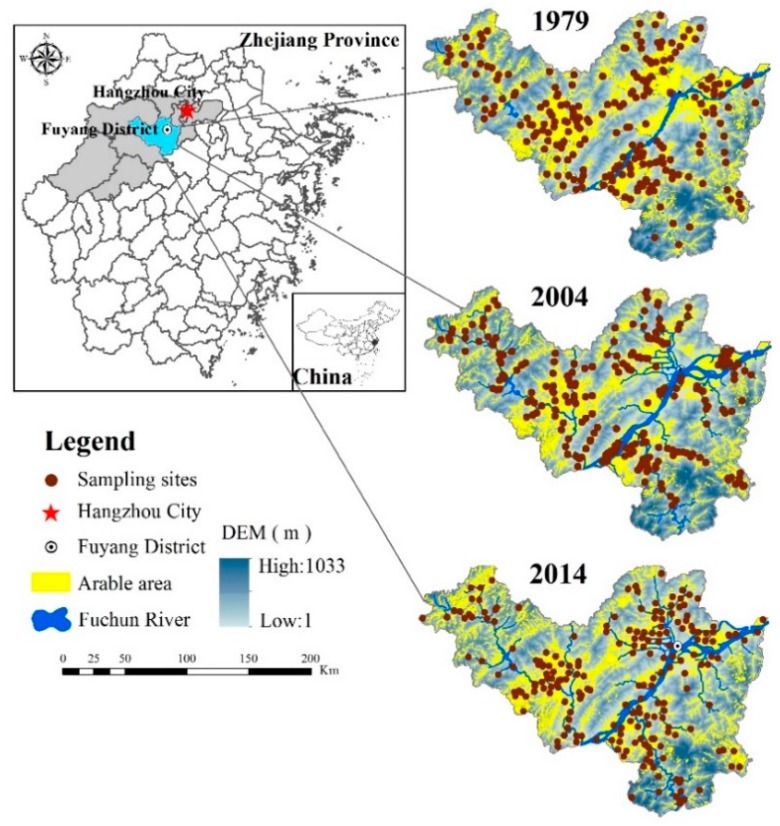
Map of the study area and locations of the sampling sites in 1979, 2004 and 2014.

To demonstrate the utility of the proposed framework, we focused on a particular study area: Fuyang District in Hangzhou City, Zhejiang Province (latitude: 29°44′45′′–30°11′58.5′′ N, longitude: 119°25′00′′–120°19′30′′ E), China. Fuyang District is only 51 km from the central city of Hangzhou, which is the key city of the Yangtze River delta economic zone, and the Fuchun River crosses through the entire district. Fuyang District covers an area of 1831 km^2^; of this area, 78.61% is covered by hills and mountains with elevations ranging from 700 m to 1500 m above sea level, and 16.36% is covered by plains and basins with elevations ranging from 6 m to 150 m above sea level. Red earths and paddy soils are the primary soil types in Fuyang District, as they account for approximately 91% of the total soil area. Among them, the red earths are mainly distributed throughout mountainous hilly areas between 200 m and 500 m above sea level, and they are characterized by a sandy and gravelly texture; in contrast, the paddy soils are mainly distributed in the valley plain area and are characterized by a clayey texture (Fuyang statistical yearbook, 1996). Fuyang District has a subtropical climate with an average temperature of 16.1 °C, and the average annual precipitation is 1414.2 mm. The agricultural land use mainly consists of rice, rape, vegetables, tea and fruit. The dominant type of cultivation system is annual double-crop rotation. Due to the geographical advantages of Fuyang District, this region has become a recreational area for the Hangzhou metropolis. In addition, the planting structure of Fuyang District, which provides large quantities of fruits and vegetables for the metropolis, is very diversified. It has been reported that extrinsic factors have played crucial roles in the soil N distribution of the district, especially with the continuous increase in anthropic interference [18]. Accordingly, because Fuyang District has experienced rapid economic growth in conjunction with the intensification of agricultural land use and the development of many recreation centers, there is an urgent need to systematically examine the spatiotemporal patterns of soil TN over the last several decades. For this purpose, we selected the arable regions of Fuyang District as our study area to explore the distribution of soil TN; specifically, the total study area was 306.72 km^2^ in 1979, 270.22 km^2^ in 2004 and 275.14 km^2^ in 2014 (Figure 1).

### 2.2. Datasets

#### 2.2.1. Soil Sample Collection and Laboratory Analysis

The following sequence of soil samples acquired from cultivated land in Fuyang District was used in this study; these samples spanned a 35-year period (Figure 1). A total of 231 soil samples from 1979 were obtained from the Second National Soil Survey of Fuyang District. The sampling locations, sampling depths, land use types, organic matter, pH, TN and other soil properties were recorded in the 1979 dataset of the Second National Soil Survey. However, because hand-held global positioning system (GPS) devices were not available in 1979, the corresponding coordinates of the sampling sites were remeasured according to the historical records of 1979. The 267 samples from 2004 and the 220 samples from 2014 were obtained from field surveys conducted to monitor the dynamics of the soil nutrients. To ensure that the soil profiles of the samples from 2004 and 2014 corresponded to those from 1979, the soil sampling locations used in 2004 and 2014 were selected to be as close to those used in 1979 as possible.

The soil samples were collected from the topsoil profile at depths of 0–20 cm, which was the same as the plowing depth, and each sample was composed of a mix of soil from five localities within a 5-m radius of a specific sampling location. Meanwhile, a hand-held GPS was used to record the geographical coordinates of every soil sampling location. The data from all sampling sites were registered to the WGS_1984_UTM_Zone_50N projection system, which is same as the other spatial maps. Then, the soil samples were all air dried for 30 days at room temperature, and the non-soil materials, such as the litter layer and stones, were removed manually. Afterward, all of the samples were crushed and passed through a 2-mm sieve and a 0.149-mm sieve to test the concentrations of soil TN using the Kjeldahl procedure [19]. Finally, due to errors in the sampling procedure and laboratory analysis, the outliers (significantly higher or lower values than the means) were excluded based on the mean ± 3SD (standard deviation).

#### 2.2.2. Environmental Variables

We employed different sets of environmental variables, namely, soil types and climatic, topographic, and vegetation variables, to predict the soil TN in each year (Table 1). These variables were selected based on their relationships with soil TN, as has been demonstrated in previous studies [11,17,20]. All of the variables were uniformly registered to the WGS_1984_UTM_Zone_50N projection system at a resolution of 30 m.

The climatic variables included the mean annual precipitation (MAP) and mean annual temperature (MAT). The Global Land Data Assimilation System (GLDAS) database, which can provide climatic and soil moisture data with a high time resolution, would be beneficial for assessing the spatiotemporal variation in soil TN [21]. In the present study, we selected the climatic dataset from the World Climate Database (1950–2010) with a high spatial resolution in consideration of the study region, which spans only 1831 km^2^. The digital elevation model (DEM) variables, including the elevation, slope and SAGA topographic wetness index (TWI), were computed using ArcGIS 10.2 and SAGA GIS software [22]; among them, the TWI was calculated based on a modified catchment due to its ability to reflect more realistic and feasible soil wetness conditions in comparison with the traditional TWI [23].

#### 2.2.3. Remote Sensing Variables

Remote sensing (RS) variables can provide detailed spectral information for large areas, and they also have the ability to reflect biotic properties; furthermore, RS variables are often economically friendly and easily accessible [24]. Thus, RS variables have become increasingly used in DSM. Among the various RS datasets, Landsat has the longest data record (i.e., since 1972) and a moderate spatial resolution. Moreover, its standard processing format can be acquired from the United States Geological Survey (USGS), and it is currently free and conveniently accessible. Therefore, RS variables have certain advantages in terms of monitoring the long-term dynamics and patterns of soil properties.

In the present study, the visible red band (B_Red_, 0.6 μm–0.7 μm) and the near-infrared band (B_NIR_, 0.7 μm–0.9 μm), which represent the vegetation growth, coverage and biomass, were collected from the Landsat images; then, the indirect effects of human activities on farmland crops were indirectly inferred [17]. To represent the vegetation cover over the study area, the normalized difference vegetation index (NDVI) was calculated using the red and near-infrared bands of the Landsat data using the formula NDVI = (B_NIR_ − B_Red_)/(B_NIR_ + B_Red_). The remotely sensed imagery was collected between July and September (i.e., during major growing seasons), and the images all had a cloud cover of less than 10%. All images were subjected to radiometric calibration and atmospheric correction by employing the fast line-of-sight atmospheric analysis of hypercubes (FLAASH) model using ENVI 5.1 software (Exelis Inc., Herndon, VA, USA).

#### 2.2.4. Other Datasets

The extents of arable land in 2004 and 2014 were acquired from the land use survey data of the Land and Resources Bureau of Fuyang. The data for the extent of arable land in 1979 were generated using a land use map with a spatial resolution of 30 m with the aid of Landsat MSS 1979 [25]. We also selected N fertilizer consumption data and crop planting structure data for the study years to better understand the soil TN variations in Fuyang District; data were collected from the Fuyang statistical yearbook and Hangzhou statistical yearbook. Moreover, the sub-regional datasets of cultivated land were obtained from the Land and Resources Bureau and the Agricultural Bureau of Fuyang.

### 2.3. Prediction Models

#### 2.3.1. Random Forest

The RF algorithm is a machine learning approach that works by building multiple regression trees and averaging the outputs of all trees to explore the relationships between the predictors and sample data. Each tree is generated based on a randomly selected subset of the original data and a subset of the predictors (with replacement) [26]. The RF algorithm estimates the general errors by using the subset of data (default: one-third of all original datasets) that are not used in the tree-building process (i.e., the out-of-bag (OOB) data).

Many previous studies have demonstrated that the RF algorithm can perform effectively in mountainous study areas because of its ability to efficiently handle nonlinear relationships between soil properties and environmental predictors [27,28]. In addition, the RF algorithm has other advantages; for example, it can provide minor tuning and a moderate flexibility regarding various types of input data [26,28]. Furthermore, it can also evaluate the relative importance (RI) of each predictor. To assess the RI, which represents the crucial role that each input predictor plays in the RF algorithm, we utilized two mechanisms: the average reduction in the accuracy, which was used to evaluate whether the effect of a variable led to any decrease, and the average reduction in the Gini index, which was used to deduce the impurity of a variable. The Gini index was used to measure the purity or uncertainty of a dataset; a smaller Gini index indicates that the purity of the dataset is higher. The calculation formula for Gini index is as follows:

(1) $$\mathrm{Gini}\;\left(p\right)=\overset{K}{\underset{k=1}{\sum }}p_{k}*\left(1-p_{k}\right)=1-\overset{K}{\underset{k=1}{\sum }}p_{k}{}^{2}$$

where *K* is the total number of classes, and $p_{k}$ is the probability that the randomly selected samples are correctly classified. Owing to the merits mentioned above, we selected the RF algorithm to map the soil TN distribution in the present study area.

The RF algorithm incorporates three parameters, namely, the number of predictors selected to build each regression tree (mtry), the number of all trees (ntree) and the minimum node size (nodesize). The strength of each tree and the correlations between trees were identified by the mtry parameter; higher mtry values indicate that each tree and the correlations between the trees will be stronger [29]. However, the RF algorithm achieves a good performance by attaining a high strength for each tree and low correlations between the trees. Therefore, to fit the RF model, ntree and nodesize were set to the default values: 500 for ntree and 5 for nodesize. Then, mtry was tuned according to the results of the training command “caret” in the R package with values of 13 for 1979, 17 for 2004, and 9 for 2014. The remaining details can be found in the article written by Breiman in 2001 [26].

To better determine the optimal model for predicting the soil TN spatial distribution and for evaluating the model performance in the present study area, we compared the RF model to another tree-based model named the BRT model, which has merits that are similar to those of the RF model. A detailed description of the BRT model is attached in the Supplementary Data. The “randomForest” and “gbm” packages in R were applied to generate the RF model and the BRT model, respectively.

#### 2.3.2. Model Validation and Uncertainty

To evaluate and compare the model prediction performances of the BRT and RF models, two frequently used indexes, namely, the root mean square error (RMSE) and the coefficient of determination (R^2^), were computed using the 10-fold cross-validation method between the predicted values and the observed soil TN values. In the 10-fold cross-validation procedure, the dataset was divided into ten subsets randomly; one subset was employed for the validation, while the other subsets were used to train the model. The algorithm was repeated 10 times, after which all of the estimates were summarized. The RMSE represents the overall performance of the prediction, while R^2^ is the proportion of the variance in the dependent variable. A higher value of R^2^ and a lower value of RMSE indicate a better model performance. The calculation formulas for the RMSE and R^2^ are attached in the Supplementary Materials. Additionally, the BRT and RF models were both generated 100 times independently, and the mean soil TN value from all 100 outputs was used as the ultimate result. The standard deviation (SD) of all prediction results was also calculated to indicate the model uncertainty.

### 2.4. Data Processing

Descriptive statistical analysis and correlation analysis were conducted in the environment of IBM SPSS Statistics 22.0 (IBM, Armonk, NY, USA), and statistical processing was performed using Excel 2007 (MICROSOFT Inc., Redmond, WA, USA). The other spatial analyses were all conducted in the environment of ArcGIS 10.2 (ESRI Inc., Redlands, CA, USA).

In the present study, Getis-Ord Gi* statistical analysis based on ArcGIS 10.2 (Esri, Redlands, CA, USA). was performed to identify and visualize hot spots and cold spots of the soil TN distribution in different periods. In contrast to the global spatial autocorrelation statistic known as Moran’s index, the Gi* statistic was designed for local autocorrelation; it calculates the raster pixels within the context of adjacent characteristics and then outputs the values of the *z*-score, *p*-value and confidence level. Hot spots are represented by high *z*-scores and low *p*-values, while cold spots are characterized by low *z*-scores and low *p*-values. Visualizing the hot spots and cold spots of the soil TN distribution is both explicitly and intuitively beneficial for the purposes of this research. Additional details can be found in the literature [30,31].

## 3. Results

### 3.1. Descriptive Statistics

The descriptive statistical results of the soil TN content in the different periods are presented in Table 2. The average TN content increased by 0.25 g kg^−1^ between 1979 and 2004 and by 0.11 g kg^−1^ between 1979 and 2014. However, the average TN content decreased by 0.14 g kg^−1^ from 2004 to 2014. We observed an increasing trend for the coefficient of variation (CV) and the standard deviation (SD) from 1979 to 2014, which could indicate that the TN content became more variable over time.

Table 3 shows the correlations between the soil TN and environmental variables. The results revealed diverse relationships between the soil TN and environmental variables during the study period. In this respect, B_Red_ and TWI were correlated negatively with TN, while the relationship between the NDVI and TN was positive. Moreover, the elevation and TWI in 1979, the TWI and B_NIR_ in 2004, and the B_NIR_ in 2014 had high correlation coefficients and significant relationships with TN. In addition, the correlations between ST and TN were significant in all periods.

### 3.2. Model Performance

**Figure 2:**
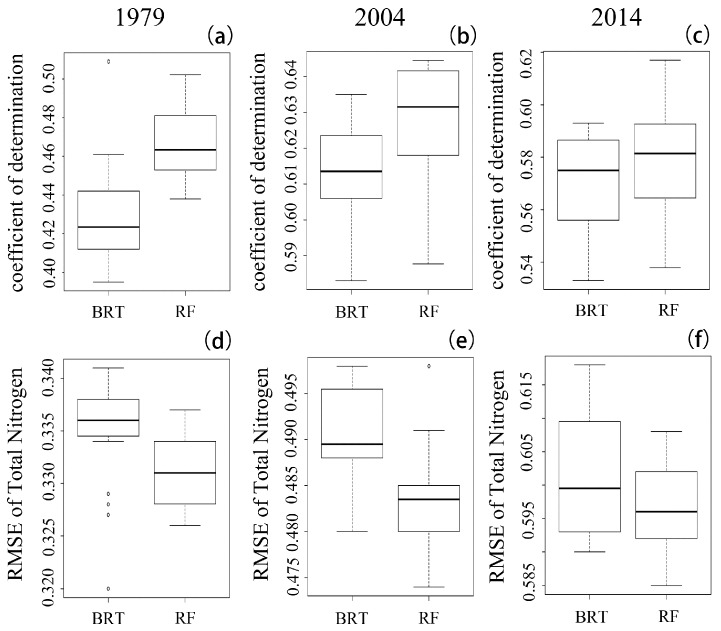
(**a**–**c**) Coefficients of determination from the 100 runs; (**d**–**f**) RMSEs from the 100 runs.

**Figure 3:**
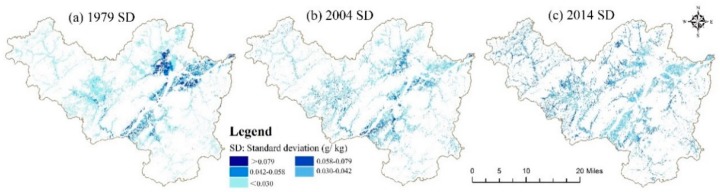
Maps of the standard deviation (SD) created using the RF model with 100 runs in (**a**) 1979, (**b**) 2004 and (**c**) 2014.

Boxplots of the BRT model, the R^2^ and the RMSE from the 100 runs are illustrated in Figure 2. The mean R^2^ and RMSE values from the 100 runs of the RF model are summarized in Table 4. The RF and BRT models had similar prediction accuracies, and they were able to explain approximately 50% of the TN variability, as demonstrated in Figure 3. In the present study, however, the RF model had a lower mean RMSE and a higher mean R^2^ than did the BRT algorithm. The BRT also showed a greater uncertainty than did the RF model concerning the variability in the RMSE. In summary, the RF model demonstrated a better performance than did the BRT model in the present study.

### 3.3. Relative Importance of Environmental Variables

**Figure 4:**
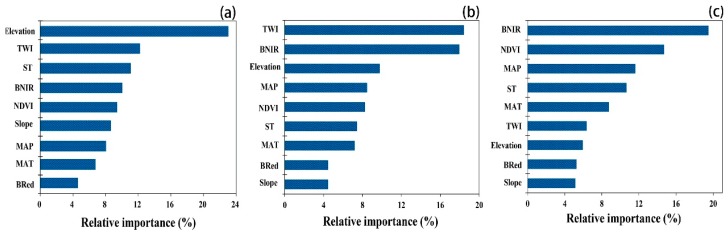
Relative importance of each predictor as determined from 100 runs of the RF model: (**a**) 1979, (**b**) 2004, and (**c**) 2014.

The numerous environmental variables used to predict the soil TN spatial distribution showed different degrees of importance in the RF model (Figure 4). 

### 3.4. Spatiotemporal Distribution of Soil TN

As presented in Table 5, we classified the soil TN content into three categories (high, medium, and low) with six levels (I: >2.0 g kg^−1^, II: 1.5–2.0 g kg^−1^, III: 1.0–1.5 g kg^−1^, IV: 0.75–1.0 g kg^−1^, V: 0.5–0.75 g kg^−1^, and VI: ≤0.5 g kg^−1^) based on the soil nutrient classification criterion of the Second National Soil Survey in China [32]. In 1979, level III encompassed the largest area (76.32%), followed by level IV (12.61%) and level II (11.07%). In 2004, level II covered the largest area (59.89%), followed by level I (59.89%) and level III (8.44%). The content level that covered the largest area in 2014 was level III (53.32%), followed by level II (43.55%).

**Figure 5:**
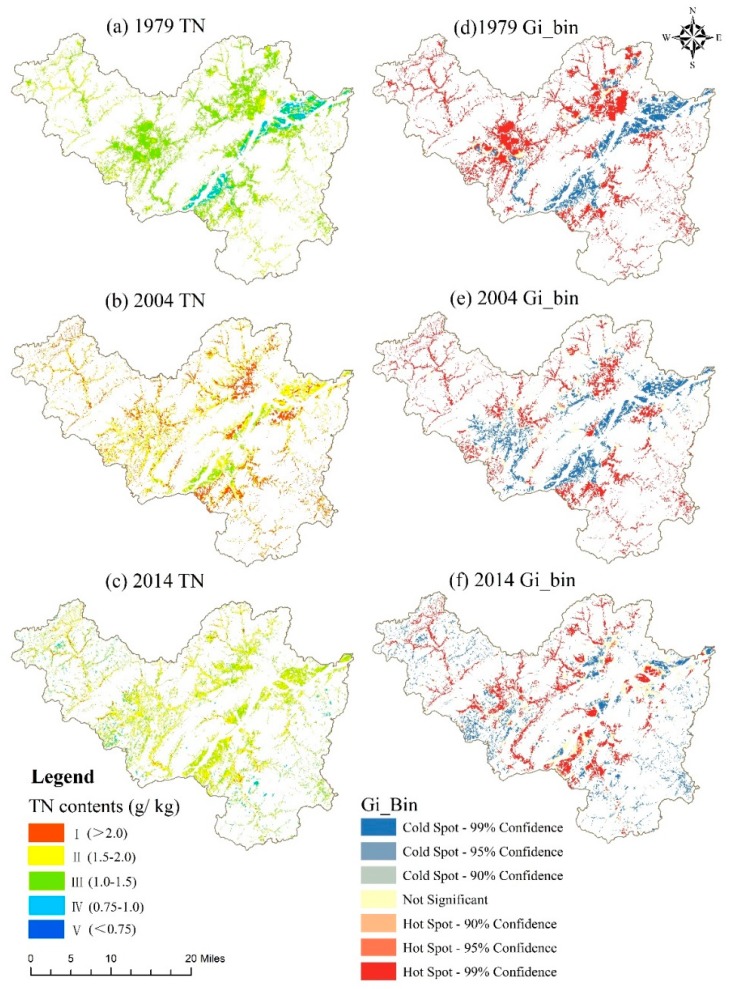
Mean TN content maps (**a**–**c**) generated by the RF model with 100 runs; hot spot and cold spot maps (**d**–**f**) in 1979, 2004 and 2014, successively.

**Figure 6:**
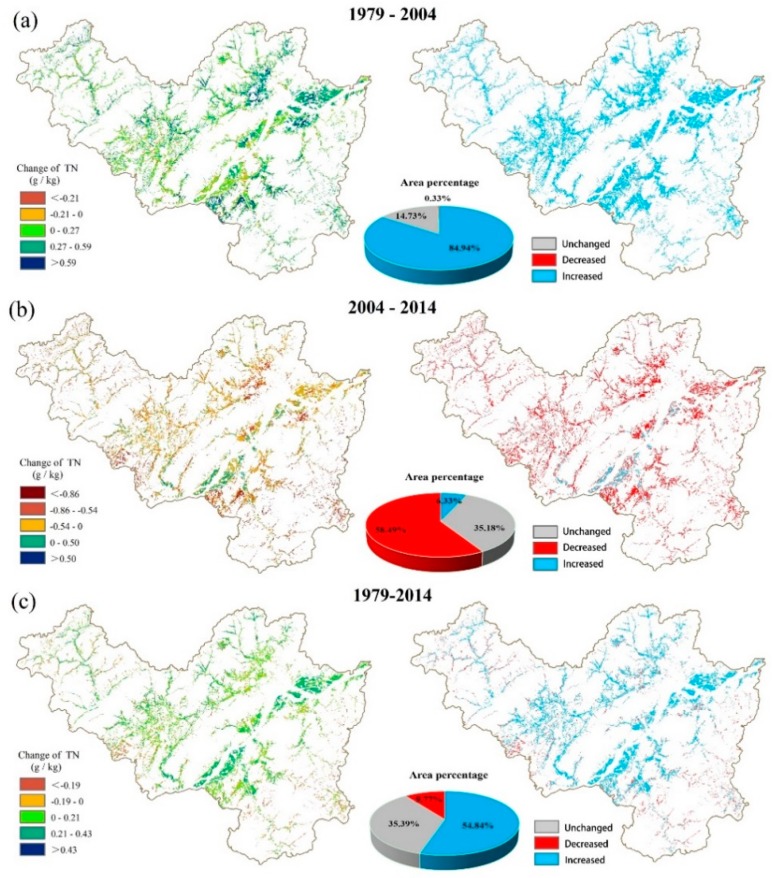
Spatiotemporal distributions of soil TN and the area percentage of TN level changes between 1979 and 2004 (**a**), 1979 and 2014 (**b**) and 2004 and 2014 (**c**).

The spatial distribution of the average soil TN content generated from 100 runs of the RF model and maps of the hot spots and cold spots in the three periods are illustrated in Figure 5. We found that the soil TN content had a homogeneous spatial distribution in 1979 (Figure 5a,d); specifically, only the northeastern and south-central areas had relatively low TN contents, while the surrounding areas had relatively high TN contents. Moreover, the soil TN content exhibited spatial patterns in 2004 similar to those in 1979 (Figure 5b,e). However, in recent decades, the soil TN showed a spatial pattern that was opposite to that observed in the earlier years (Figure 5c,f). The changes in the soil TN contents during the different periods are illustrated in Figure 6. In the present study region, the ratios of the area with an increased TN content were 84.94% and 54.84% during 1979–2004 and 1979–2014, respectively.

**Figure 7:**
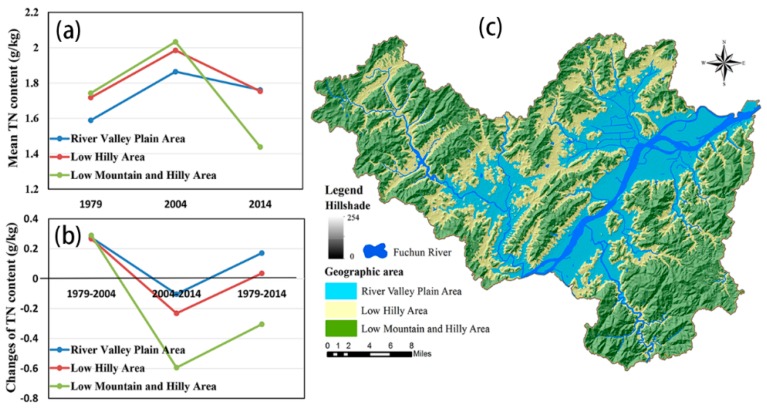
The average TN (**a**) and the changes in TN (**b**) in three geographic areas (**c**) were also analyzed in the present study to further understand the topographic effects on the spatiotemporal distributions of soil TN in cultivated lands.

**Figure 8:**
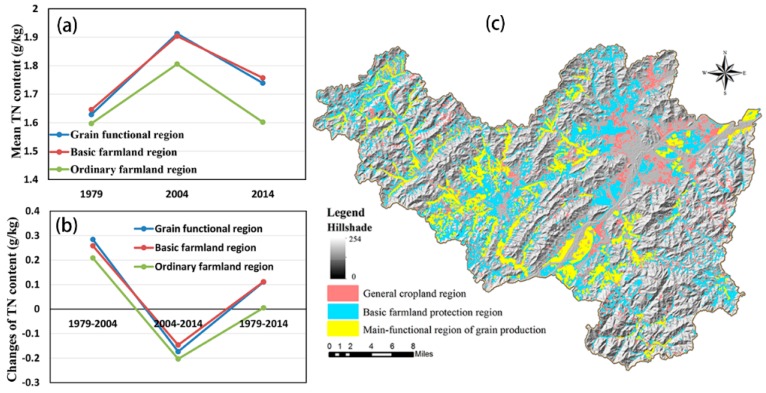
The average TN (**a**) and the changes in TN (**b**) in three farmland regions (**c**) were also analyzed in the present study to further understand the effects of land management on the spatiotemporal distributions of soil TN in cultivated lands.

To further comprehend the effects of the geographic and RS variables on the soil TN distribution, we also analyzed the spatiotemporal changes in soil TN in different geographic areas and different cultivated land protection areas in Fuyang (Table 6, Figure 7 and Figure 8). We found that the mean TN content increased as the altitude increased in the early years. However, in 2014, the river valley plain areas had a higher mean TN content than did the other areas, which contrasted with the previous trend (Figure 7a). Additionally, the ordinary farmland regions had a generally lower mean TN content than did the other regions in all of the investigated periods (Figure 8a). Figure 8b shows that the TN content in the grain functional regions and in the basic regions increased more than did the TN content in the ordinary farmland regions. The TN content in the river valley plain areas also had a higher increasing trend (Figure 7b). It is worth noting that the TN contents in the low mountains and hilly areas as well as in the ordinary farmland regions showed more obvious decreasing trends (Figure 7b and Figure 8b).

## 4. Discussion

### 4.1. Model Performance

In the present study, the RF model demonstrated good performance in mapping the soil TN distribution. Jeong et al. also found that the RF model showed lower variability in the RMSE and R^2^ values when predicting soil N and other soil properties [27]. Although there were different uncertainty factors, such as differences in the sampling strategy in addition to experimental errors and model running errors [11], which may have influenced the soil TN analysis, the RF model proved to be stable in terms of predicting soil TN; this stability was supported by its low SD values among the performance results (Table 4, Figure 3). Because human activities (e.g., fertilization, tillage and residue treatments) substantially influence the distribution of soil nutrients [10] and because complex relationships exist between environmental variables and soil nutrients, machine learning algorithms that can efficiently handle nonlinear relationships are becoming more popular in terms of predicting the distribution of soil properties. Based on its good model performance and other advantages, such as the relatively small number of parameters and the flexibility regarding various types of input datasets, the RF model might have the potential to map the soil TN distribution in the present study region.

### 4.2. Roles of Environmental Factors

The spatial distributions of the soil properties in arable land are controlled by various environmental factors, including natural characteristics (e.g., the geography, climate and soil type) and RS variables. In the present study, the soil type always exhibited a significant correlation with the soil TN content; among for the climatic variables, the MAP had a greater influence on the TN than did the MAT. Guntiñasaaba et al. found that the soil moisture and temperature have significant effects on net soil TN mineralization, thereby indicating the potential of the soil moisture and climatic variables for mapping the soil TN distribution [33]. Moreover, two topographic variables (i.e., the elevation and TWI) were identified as the most influential factors for predicting the soil TN content, especially in the early years. The elevation could play a central role in the spatial pattern of soil TN because it adjusts the microclimate of cultivated land and affects the microbial activities related to decomposition and transformation of soil TN [34]. The TWI could also have an impact on the soil TN distribution, as it pertains to the potential of a region being wet [28].

The RS variables, including B_NIR_ and NDVI, were significantly correlated with the soil TN concentration (Table 3), and these variables performed the best (Figure 4) in predicting the soil TN in both 2004 and 2014; these results are similar to those presented by previous studies [11,27]. The B_NIR_ and NDVI were the key predictors for mapping the TN distribution owing to their abilities to effectively reflect the vegetation coverage and biomass in the study area [17]. Previous studies have confirmed that the TN content is strongly affected by land use patterns [35]. The NDVI, which was synthesized through a calculation involving B_red_ and B_NIR_, can effectively represent the land use conditions, and thus, it demonstrates significant effects on variations in soil TN. Wang et al. (2012) and Yang et al. also reported the same findings in their studies related to mapping the TN and soil organic carbon distributions by using RS variables [28,36]. However, we observed that soil TN had a weaker correlation with B_Red_ than with the climate and topography variables (Table 3); moreover, B_Red_ performed poorly when it was used to predict soil TN. This finding is consistent with the results of Yang et al. [37], who observed that the vegetable distribution was primarily dependent on the distribution of the climate and topography; thus, the role of B_Red_ would be weakened by climatic and topographic factors. Furthermore, the reflectance spectrum of the plant leaf and canopy can be used to indicate land use patterns, such as cropping structures [28]; therefore, the RS variables played an important role in terms of controlling the soil TN conditions.

From the perspective of their various roles, the natural characteristics, such as the geographic variables, played crucial roles in terms of controlling the soil TN distribution in the early years. In terms of their importance, the RS variables rose to second place in 2004, after which they became the most crucial factors in 2014 (Figure 4). The changes in the relative importance of the variables showed that the RS indexes became increasingly important in controlling the soil TN distribution patterns. RS variables are being increasingly utilized in DSM due to their ability to respond to extremely complex and heterogeneous landscapes; further, these variables can be used to infer land use patterns caused by human interference on farmland [24,38], which could also explain their increasingly important roles observed in the present study. The information about cultivated land provided by RS variables can not only indicate the direct crop growth properties but also demonstrate the indirect impacts of agricultural management practices concerning crops. Similar results were reported in previous studies [18], e.g., extrinsic factors became increasingly important with regard to their effect on the soil TN distribution. In the future, the mapping of soil TN in cultivated land should consider the use of RS variables, especially in areas with relatively complex topography.

### 4.3. Spatiotemporal Distributions of Soil TN

#### 4.3.1. Spatial Patterns of Soil TN

Similar spatial patterns of soil TN were found in 1979 and 2004, and the mean TN content increased as the altitude increased in the early years. These findings could be partly due to the high background values of TN in these mountainous areas and partly because many farmers were located in remote mountainous areas with a high per capita of arable land. Additionally, high quantities of animal manure were applied to the arable land, and straws were returned to the arable land in Fuyang (Fuyang Statistical Yearbook, 1996).

However, in recent decades, the soil TN distribution showed an opposite spatial pattern compared with that observed in the early years. For example, the river valley plain areas had a higher mean TN content in contrast to the previous trend, as shown in Figure 7. According to the Fuyang statistical yearbook, the central and northeastern plain areas near the Fuchun River of Fuyang District have been the main cash crop planting areas since the 2000s, because these areas are closer to the downtown region, which has a greater demand for cash crops (e.g., vegetables). Consequently, additional N fertilizers were applied to the arable land to ensure high production. A previous study [39] also showed that the land in which cash crops, especially vegetables, were planted had a higher TN content. Similarly, we observed that the mean TN contents were lower in ordinary farmland regions than in grain functional regions and basic farmland regions in all periods. According to the defined rules of cultivated land, the ordinary farmland regions had a poor quality and were not conducive to farming; these reasons may explain why the TN content was lower in these regions.

#### 4.3.2. Temporal Changes in Soil TN

**Figure 9:**
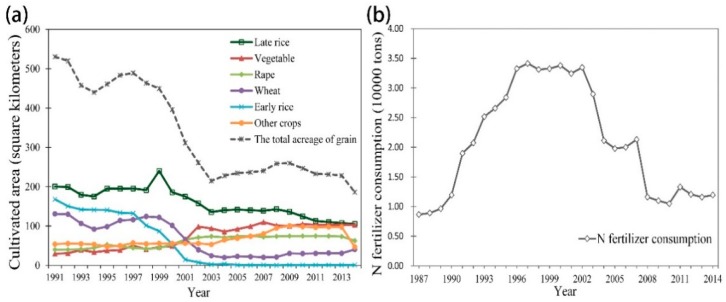
The crop planting structure in Fuyang District from the 1990s to the 2010s (**a**) and the content of nitrogen fertilizer consumption in Fuyang District from the 1980s to the 2010s (**b**).

In the present study region, the soil TN content showed an increasing trend in the early periods. Similar results have been observed in many other areas in former studies throughout China [6,40]. Li et al. reported that the soil TN content increased by 27.27% from 1981 to 2012 in Renshou County, which is located in the hilly region of the mid-Sichuan Basin in southwestern China [18]. Jiang et al. reported that the average soil TN content significantly increased by 0.08 g kg^−1^ in the farmland of Yangon County, which is located in the ecological economic area of Poyang Lake in China [35]. As mentioned above, the land use pattern factors became more important in terms of their influence on the soil TN distribution, and we found that the N fertilizer inputs and crop planting structure played critical roles on the amount of soil TN in the present study area; these results were supported by the results of previous studies [18,39]. The Household Responsibility System (HRS), which was initiated in the late 1970s in China, greatly improved farmer enthusiasm for planting [41]. The collective farming system was converted into individually owned family farms by the HRS policy, and this change affected the nutrient conditions of the arable land [6]. Farmers gradually increased the amount of fertilizers they applied to arable land to achieve higher yields. According to the N fertilizer consumption data collected from the Fuyang and Hangzhou statistical yearbooks (1987–2014), N fertilizer consumption in Fuyang showed an increasing trend from the earliest study years to the 2000s (Figure 9). A previous study reported that fertilizer application could significantly improve the soil TN content after ten years based on long-term N experiments in southern China [40]. Therefore, N fertilizer inputs have become a main factor that can improve the soil TN content in the present study region.

Another important factor that influenced the spatiotemporal distribution of soil TN was the crop planting structure. On the one hand, after the HRS policy was implemented in China in the late 1970s, farmers had the authority to manage their crops, and many of them chose to plant cash crops to increase their income [6]. On the other hand, the winter crop planting structure in Fuyang District changed in the 1990s. As illustrated by the crop planting structure data of Fuyang District collected from the Fuyang and Hangzhou statistical yearbooks (1991–2014) (Figure 9), the proportion of the planted area of grain crops decreased by 65% from 1991 to 2014. Among them, the proportions of late rice, early rice and wheat decreased by 47.5%, 99.48% and 69.23%, respectively. In contrast, the planting ratios of vegetable and rape increased by 2.3 times and 0.57 times, respectively, from 1991 to 2014. The changed planting structure, which included more cash crops, required more corresponding nutrient inputs than did the grain crops [18]. Therefore, the planting structure became another important factor that influenced the amount of soil TN in Fuyang District. In addition, green manure planting and straw returning in Fuyang also affected the increasing tendency of the soil TN content.

The results showed that the mean soil TN content in the arable land of Fuyang District decreased by 58.49% between 2004 and 2014 (Figure 6). The main causes of this TN reduction might have been the policy promoting STFFT in vast areas of China; this policy was implemented in the 2000s, and it aimed to reduce irrational fertilization. The STFFT provided a more moderate and balanced fertilization scheme for farmers based on the actual nutrient status of a field. Listed as a demonstration county of the national STFFT by the Ministry of Agriculture of China starting in the early 2000s, Fuyang District has actively promoted the STFFT. The entire region of Fuyang District has applied the STFFT over an area of 50,700 ha; specifically, as of 2014, the region has 278 core demonstration zones, it has promoted 10,513 tons of formulated fertilizers, and it has saved 1124 tons of fertilizers (Fuyang Statistical Yearbook, 2014). We can also visually conclude that the N fertilizer consumption has significantly decreased since the 2000s (Figure 9), which is in agreement with the mapping results of soil TN that demonstrated a decreasing trend between 2004 and 2014 in Fuyang (Figure 6).

**Figure 10:**
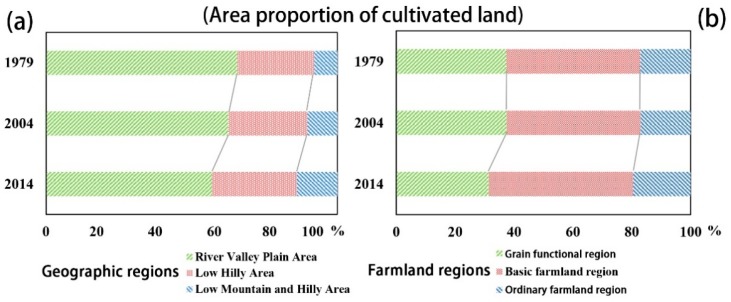
Area proportion of cultivated land under different geographic areas (**a**) and farmland regions (**b**) in different periods.

In addition, the area of cultivated land distributed in the river valley plain areas and in the grain functional regions gradually decreased; this pattern contrasted with the increasing trend shown in the low mountains and hilly areas as well as in the ordinary farmland regions (Figure 10). The aforementioned changes in the proportion of cultivated land distribution indicate that the quality of cultivated land in Fuyang exhibited a decreasing trend. A previous study demonstrated that large amounts of high-quality fertile farmland were occupied by urbanization and that most of the newly added farmland was located in areas with higher elevations, inconvenient transportation systems and poor infrastructure; these conditions hindered farmers from farming and managing these fields [42]. These might be the reasons why the TN contents in the low mountains and hilly areas as well as in the ordinary farmland regions showed more obvious downward trends (Figure 7 and Figure 8). In addition to the site-specific conditions of the arable land, including the convenience of transportation, the availability of irrigation, the patch size and the farming distance [43] as well as the variable size of the proportion of farmers in the population, the income of farmers and the popularity of field mechanization practices [44] have also had increasing roles in their effects on the soil TN distribution in both space and time.

## 5. Conclusions

In this study, the RF model was used to map the spatial distribution patterns of soil TN, and the lower SD and RMSE values as well as higher R^2^ values indicated that the RF model was stable and effective in terms of mapping the soil TN distribution in the present study region. We also found that various environmental variables played different roles regarding their controls on the soil TN conditions over time. Geographic and RS variables were identified as the indexes with a higher relative importance than that of any other variable in the study. The main factors that influenced the soil TN distribution shifted from natural characteristics in the early years to the RS variables in more recent decades. Regarding the spatiotemporal patterns of soil TN, the TN content exhibited an increasing trend in the early decades, while it decreased between 2004 and 2014. The application of N fertilizer and crop planting structure, which were caused by the STFFT as well as farmland protection policies, played the most important roles in terms of influencing the dynamics of the soil TN content in the study area. Moreover, the site-specific conditions of the cultivated land also had an increasing role in affecting the spatiotemporal TN patterns of cultivated land. In the future, nutrient balance management policies should be implemented based on the local conditions.

## Figures and Tables

**Table 1:** Description of the environmental variables utilized for predicting soil TN.

Variables	Name	Unit	Scale	Source
Soil Type	ST	-	1:50,000	Digitized soil type map of Fuyang District
Climate	MAP	mm	1000 m	World climate database (1950–2010)
	MAT	°C		(http://www.worldclim.org/)
Topography	Elevation	M	30 m	DEM data, Geospatial Data Cloud site, Chinese Academy of Sciences (http://www.gscloud.cn)
	Slope	°		
	TWI	-		
Remote Sensing	B_Red_	-	79 m & 30 m	Landsat 3 MSS on 5 August 1979 (79 m); Landsat 5 TM on 26 July 2004 (30 m); Landsat 8 OLI on 22 July 2014 (30 m); USGS (https://glovis.usgs.gov/)
	B_NIR_	-		
	NDVI	-		

**Table 2:** Summary of the statistics of the soil TN datasets in Fuyang District.

Year	Number	Min (g/kg)	Max (g/kg)	Mean (g/kg)	SD (g/kg)	CV (%)
1979	231	0.60	2.50	1.65	0.36	21.82
2004	267	0.44	3.70	1.90	0.56	29.47
2014	220	0.40	3.50	1.76	0.69	47.26

SD: Standard deviation; CV: Coefficient of variation.

**Table 3:** Correlations between the TN concentration and environmental variables.

Year	Elevation	Slope	TWI	MAT	MAP	ST	B_Red_	B_NIR_	NDVI
1979	0.42 ^b^	0.24 ^a^	−0.31 ^b^	0.18	0.36 ^b^	0.37 ^b^	−0.01	0.21	0.15
2004	0.29 ^a^	−0.06	−0.41 ^b^	−0.14	0.27 ^b^	0.24 ^a^	−0.31 ^b^	−0.33 ^b^	0.18
2014	−0.21 ^a^	−0.21 ^a^	−0.29	0.22	−0.32 ^b^	0.25 ^a^	−0.11	0.44 ^b^	0.38 ^b^

^a^ Significant correlation at the level of *p* < 0.05; ^b^ Significant correlation at the level of *p* < 0.01.

**Table 4:** Summary statistics of the RF performance in predicting the soil TN distribution with 100 runs.

Year	Index	Min	Max	Mean	SD
1979	RMSE	0.33	0.34	0.3312	0.0039
	R^2^	0.44	0.50	0.4677	0.0180
2004	RMSE	0.47	0.50	0.4833	0.0054
	R^2^	0.59	0.64	0.6286	0.0149
2014	RMSE	0.59	0.61	0.5968	0.0067
	R^2^	0.54	0.62	0.5796	0.0194

**Table 5:** Basic nutrients grades of soil TN.

Classification	Level	TN	1979		2004		2014	
		(g/kg)	Area (ha)	Percentage (%)	Area (ha)	Percentage (%)	Area (ha)	Percentage (%)
High	I	>2.0	0	0	8559	31.67	201	0.73
	II	1.5–2.0	3395	11.07	16,183	59.89	11,982	43.55
Medium	III	1.0–1.5	23,409	76.32	2280	8.44	14,671	53.32
	IV	0.75–1.0	3868	12.61	0	0	620	2.22
Low	V	0.5–0.75	0	0	0	0	40	0.2
	VI	≤0.5	0	0	0	0	0	0

**Table 6:** Details of the farmland regions and geographic regions in Fuyang District.

Region	Sub-Region	Characteristics
Farmland regions	Grain functional region	Relatively concentrated contiguous and well-established rice cultivation areas delineated by the Bureau of Agriculture
	Basic farmland region	Higher-quality arable areas delineated by the Land and Resources Bureau. The scope of this area did not include the aforementioned grain functional region in the present study
	Ordinary farmland region	The remainder of cultivated land areas with lower-quality characteristics
Geographic regions	River falley plain region	Mainly including valleys and river plains with a relative height ranging from 0 to 50 m
	Low hilly region	With a relative height ranging from 50 to 150 m
	Low mountain and hilly region	Mainly including the surrounding low mountains and hilly areas with a relative height exceeding 150 m

## Appendix A

**BRT:**

The boosted regression tree (BRT) is an ensemble tree model used to improve the model prediction performance by fitting multiple different models and combining them for an enhanced prediction [45]. Two typical algorithms are integrated within the BRT model: the regression tree and the boosting technique. Regression trees are derived from the classification and regression tree (CART), which connects the response variable to quantities of predictors through recursive binary splits [46]. The binary split continues to lend itself to its own result until it reaches predefined termination requirements. The boosting technique is an algorithm employed to improve the accuracy of the regression tree; it resembles a model averaging approach, in which the outputs of several competing models are merged. However, the difference between boosting and model averaging is that boosting is a forward and stage-wise sequential algorithm. In a forward and stage-wise procedure, a subset of training data is stochastically selected to iteratively fit new tree models. The BRT algorithm was utilized based on a stochastic gradient boosting procedure, in which regression trees were grown iteratively by recursive binary splits until the minimum deviation was reached. As mentioned above, the final fitted BRT model exhibited a good prediction performance with less over-fitting and faster convergence. In the present study, the BRT model was implemented in the R environment with the “gbm” version 2.1 package.

Four parameters, namely, the learning rate (LR), tree complexity (TC), bag fraction (BF) and number of trees (NT), must be pre-tuned by the user. The LR indicates the contribution of each tree to the ultimate fitted model. The TC (the number of nodes in a tree) regulates the size of the trees and whether the interactions between variables should be taken into account. The BF represents the proportion of datasets applied in each model, while the NT value is determined by both the LR and the TC [45]. The optimal parameter combination can provide the minimum predictive deviance. To determine the optimal parameters and acquire a better prediction performance with the minimum predictive deviance, various combinations of LR, TC, BF and NT were tested in the present study. The final optimal values of LR, TC, BF and NT were set as 0.0025, 9, 0.75 and 1000, respectively, by the 10-fold cross validation method. A detailed description can be found in the working guide to boosted regression trees [45].

**RMSE and R^2^:**

The RMSE and R^2^ values were calculated as follows:

$$\mathrm{RMSE}=\sqrt{\frac{1}{n}\sum_{i=1}^{n}{\left(P_{i}-O_{i}\right)}^{2}}$$

$$R^{2}=\frac{\sum_{i=1}^{n}{\left(P_{i}-O_{i}\right)}^{2}}{\sum_{i=1}^{n}{\left(O_{i}-\bar{O}\right)}^{2}}$$

where $P_{i}$, $O_{i}$ and $\bar{O}$ are the predicted values, the observed values, and the mean value, respectively, of all of the observations at site *i*, and *n* is the quantity of samples.

## References

[1] Powlson D.S., Gregory P.J., Whalley W.R., Quinton J.N., Hopkins D.W., Whitmore A.P., Hirsch P.R., Goulding K.W.T. (2011). Soil management in relation to sustainable agriculture and ecosystem services. Food Policy.

[2] Smith P., Cotrufo M.F., Rumpel C., Paustian K., Kuikman P.J., Elliott J.A., Mcdowell R., Griffiths R.I., Asakawa S., Bustamante M. (2015). Biogeochemical cycles and biodiversity as key drivers of ecosystem services provided by soils. Soil.

[3] Stevens C.J., Dise N.B., Mountford J.O., Gowing D.J. (2004). Impact of nitrogen deposition on the species richness of grasslands. Science.

[4] Wardle D.A. (1992). A comparative assessment of factors which influence microbial biomass carbon and nitrogen levels in soil. Biol. Rev..

[5] Bronson K.F., Zobeck T.M., Chua T.T., Acostamartinez V., Van Pelt R.S., Booker J.D. (2004). Carbon and nitrogen pools of southern high plains cropland and grassland soils. Soil Sci. Soc. Am. J..

[6] Liu X.M., Zhang W.W., Zhang M.H., Ficklin D.L., Fan W. (2009). Spatio-temporal variations of soil nutrients influenced by an altered land tenure system in China. Geoderma.

[7] Velthof G.L., Lesschen J.P., Webb J., Pietrzak S., Miatkowski Z., Pinto M., Kros J., Oenema O. The impact of the Nitrates Directive on nitrogen emissions from agriculture in the EU-27 during 2000–2008. Proceedings of the Conference on Computer Vision & Pattern Recognition Workshop.

[8] Chen T.E., Zhao C.J., Chen L.P., Chen H. (2008). Research on component-oriented decision-making support platform of soil testing and formulated fertilization. Appl. Res. Comput..

[9] Kong X. (2014). China must protect high-quality arable land. Nature.

[10] Zhao Y., Wang M., Hu S., Zhang X., Ouyang Z., Zhang G., Huang B., Zhao S., Wu J., Xie D. (2018). Economics- and policy-driven organic carbon input enhancement dominates soil organic carbon accumulation in Chinese croplands. Proc. Natl. Acad. Sci. USA.

[11] Wang S., Adhikari K., Wang Q., Jin X., Li H. (2017). Role of environmental variables in the spatial distribution of soil carbon (C), nitrogen (N), and C:N ratio from the northeastern coastal agroecosystems in China. Ecol. Indic..

[12] Holmberg M., Forsius M., Starr M., Huttunen M. (2006). An application of artificial neural networks to carbon, nitrogen and phosphorus concentrations in three boreal streams and impacts of climate change. Ecol. Model..

[13] Jeong G., Choi K., Spohn M., Park S.J., Huwe B., Ließ M. (2017). Environmental drivers of spatial patterns of topsoil nitrogen and phosphorus under monsoon conditions in a complex terrain of South Korea. PLoS ONE.

[14] Guan F., Xia M., Tang X., Fan S. (2017). Spatial variability of soil nitrogen, phosphorus and potassium contents in Moso bamboo forests in Yong’an City, China. Catena.

[15] Wang K., Zhang C., Li W. (2013). Predictive mapping of soil total nitrogen at a regional scale: A comparison between geographically weighted regression and cokriging. Appl. Geogr..

[16] Zhang G.L., Liu F., Song X.D. (2017). Recent progress and future prospect of digital soil mapping: A review. J. Integr. Agric..

[17] Mcbratney A.B., Santos M.L.M., Minasny B. (2003). On digital soil mapping. Geoderma.

[18] Li Q., Luo Y., Wang C., Li B., Zhang X., Yuan D., Gao X., Zhang H. (2016). Spatiotemporal variations and factors affecting soil nitrogen in the purple hilly area of Southwest China during the 1980s and the 2010s. Sci. Total Environ..

[19] Page A.L. (1982). Methods of Soil Analysis. Part 2. Chemical and Microbiological Properties.

[20] Heung B., Hodúl M., Schmidt M.G. (2017). Comparing the use of training data derived from legacy soil pits and soil survey polygons for mapping soil classes. Geoderma.

[21] Zawadzki J., Kȩdzior M.A. (2014). Statistical analysis of soil moisture content changes in Central Europe using GLDAS database over three past decades. Open Geosci..

[22] Conrad O., Bechtel B., Bock M., Dietrich H., Fischer E., Gerlitz L., Wehberg J., Wichmann V., Böhner J. (2015). System for Automated Geoscientific Analyses (SAGA) v. 2.1.4. Geosci. Model Dev. Discuss..

[23] Boehner J., Koethe R., Conrad O., Gross J., Ringeler A., Selige T. (2002). Soil Regionalisation by Means of Terrain Analysis and Process Parameterization.

[24] Grunwald S. (2009). Multi-criteria characterization of recent digital soil mapping and modeling approaches. Geoderma.

[25] Zhong T., Huang X., Zhang X., Wang K. (2011). Temporal and spatial variability of agricultural land loss in relation to policy and accessibility in a low hilly region of southeast China. Land Use Policy.

[26] Breiman L. (2001). Random Forests. Mach. Learn..

[27] Jeong G., Oeverdieck H., Park S.J., Huwe B., Ließ M. (2017). Spatial soil nutrients prediction using three supervised learning methods for assessment of land potentials in complex terrain. Catena.

[28] Yang R.M., Zhang G.L., Liu F., Lu Y.Y., Yang F., Yang F., Yang M., Zhao Y.G., Li D.C. (2016). Comparison of boosted regression tree and random forest models for mapping topsoil organic carbon concentration in an alpine ecosystem. Ecol. Indic..

[29] Peters J., Verhoest N., Samson R., Boeckx P., De Baets B. (2008). Wetland vegetation distribution modelling for the identification of constraining environmental variables. Landsc. Ecol..

[30] Mitchel A. (2005). The ESRI Guide to GIS Analysis, Volume 2: Spatial Measurements and Statistics.

[31] Arthur G. (2010). The analysis of spatial association by use of distance statistics. Geogr. Anal..

[32] Zhejiang Soil Survey Office (1993). Zhejiang Soil Species.

[33] Guntiñasaaba M.E., Leirós M.C., Trasar-Cepeda C., Gil-Sotres F. (2012). Effects of moisture and temperature on net soil nitrogen mineralization: A laboratory study. Eur. J. Soil Biol..

[34] Wang S., Zhuang Q., Wang Q., Jin X., Han C. (2017). Mapping stocks of soil organic carbon and soil total nitrogen in Liaoning Province of China. Geoderma.

[35] Jiang Y., Rao L., Sun K., Han Y., Guo X. (2018). Spatio-temporal distribution of soil nitrogen in Poyang lake ecological economic zone (South-China). Sci. Total Environ..

[36] Wang S., Wang X., Ouyang Z. (2012). Effects of land use, climate, topography and soil properties on regional soil organic carbon and total nitrogen in the Upstream Watershed of Miyun Reservoir, North China. J. Environ. Sci..

[37] Yang R., Rossiter D.G., Liu F., Lu Y., Yang F., Yang F., Zhao Y., Li D., Zhang G. (2015). Predictive mapping of topsoil organic carbon in an alpine environment aided by Landsat TM. PLoS ONE.

[38] Mulder V.L., de Bruin S., Schaepman M.E., Mayr T.R. (2011). The use of remote sensing in soil and terrain mapping—A review. Geoderma.

[39] Liu L.L., Zhu Y., Liu X.J., Cao W.X., Xu M., Wang X.K., Wang E.L. (2014). Spatiotemporal changes in soil nutrients: A case study in Taihu region of China. J. Integr. Agric..

[40] Wang J., Zhu B., Zhang J., Müller C., Cai Z. (2015). Mechanisms of soil N dynamics following long-term application of organic fertilizers to subtropical rain-fed purple soil in China. Soil Biol. Biochem..

[41] Lin J.Y. (1987). The household responsibility system reform in China: A peasant’s institutional choice. Am. J. Agric. Econ..

[42] Lin L., Ye Z., Gan M., Shahtahmassebi A.R., Weston M., Deng J., Lu S., Wang K. (2017). Quality perspective on the dynamic balance of cultivated land in Wenzhou, China. Sustainability.

[43] Li W., Wang D., Li H., Liu S. (2017). Urbanization-induced site condition changes of peri-urban cultivated land in the black soil region of northeast China. Ecol. Indic..

[44] Tittonell P., Muriuki A., Shepherd K.D., Mugendi D., Kaizzi K.C., Okeyo J., Verchot L., Coe R., Vanlauwe B. (2010). The diversity of rural livelihoods and their influence on soil fertility in agricultural systems of East Africa—A typology of smallholder farms. Gallimard.

[45] Elith J., Leathwick J.R., Hastie T. (2008). A working guide to boosted regression trees. J. Anim. Ecol..

[46] Breiman L., Friedman J.H., Olshen R.A., Stone C.J. (1984). Classification and Regression Trees.

